# Comparing the Effectiveness of Group Discussion and SMS-Based Education on Nutritional Status, Physical Activity, and Blood Cholesterol Levels in Women with Elevated Cholesterol: A Randomized Controlled Trial

**DOI:** 10.5812/ijem-160891

**Published:** 2025-07-31

**Authors:** Hossein Ashtarian, Neda Yavari, Behrooz Hamzeh, Afshin Almasi

**Affiliations:** 1 Department of Health Education and Promotion, Faculty of Health, Kermanshah University of Medical Sciences (KUMS), Kermanshah, Iran; 2 Department of Biostatics, Faculty of Health, Kermanshah University of Medical Sciences (KUMS), Kermanshah, Iran

**Keywords:** Dietary Habits, Physical Activity, Blood Cholesterol, Education, SMS, Group Discussion

## Abstract

**Background:**

Given the significant contribution of dyslipidemia to cardiovascular disease (CVD) risk in women, effective educational interventions targeting lifestyle modifications are crucial for disease prevention and management.

**Objectives:**

The present study was conducted with the aim of comparing the effectiveness of group discussion and short message service (SMS)-based education in improving nutrition, physical activity, and cholesterol levels in women with high blood cholesterol.

**Methods:**

A randomized trial was conducted with 165 women aged 30 - 59, recruited from two urban health centers in Kermanshah, Iran. The study included participants with borderline high cholesterol levels (200 - 239 mg/dL) who were randomly assigned to one of three groups (n = 55 each): An SMS-based education group receiving text messages on nutrition and physical activity, a group discussion intervention with facilitated sessions on lifestyle modification, or a control group that received standard care without additional educational components. Participants were randomly assigned to one of three groups using permuted block randomization [block sizes of (e.g. 6, 9)], with allocation concealment ensured by sealed, opaque envelopes. With a statistical power of 80% and a confidence level of 95%, the minimum required sample size was calculated. Questionnaires were administered to evaluate dietary and physical activity habits before and three months after the interventions. Blood samples were also obtained at these time points. Statistical analysis was performed using SPSS version 16. The chi-square, Wilcoxon signed-rank, and Kruskal-Wallis tests were used for baseline, within-group, and between-group comparisons, respectively. A P-value of less than 0.05 was considered statistically significant.

**Results:**

The two intervention groups showed a statistically significant increase in the mean score of nutritional status and physical activity and a decrease in the level of blood cholesterol after the intervention, compared to the control group (P < 0.001). There was no significant difference between the SMS and discussion groups in terms of physical activity and cholesterol levels, but the nutritional status score in the discussion group was significantly higher than in the SMS group.

**Conclusions:**

Both group discussion and SMS interventions can be effective strategies for promoting healthy lifestyle changes in women with high cholesterol. Group discussions appear to have a broader impact, as they also improve nutritional status. Healthcare providers can consider incorporating these methods into their interventions to help women manage their cholesterol levels and improve their overall health.

## 1. Background

Cardiovascular diseases (CVDs) are a primary cause of worldwide morbidity and mortality, and elevated cholesterol levels are recognized as a significant modifiable risk factor in women. Borderline high cholesterol, defined as levels between 200 and 239 mg/dL, presents a critical juncture for intervention to prevent progression to more severe hyperlipidemia and subsequent cardiovascular events (1, 2). Lifestyle modifications, such as dietary adjustments and increased physical activity, constitute the cornerstone of cholesterol management and CVD risk reduction (3). However, effectively translating this knowledge into sustained behavior change, especially among women, remains a significant public health challenge (4, 5).

Traditional health education approaches, such as face-to-face counseling, have demonstrated some limited efficacy in promoting lifestyle changes. In a meta-analysis study examining the effect of behavioral counseling on 15 randomized controlled trials, the results showed that these programs reduced LDL but had no significant effect on HDL and total cholesterol (6). Evidence suggests that different educational intervention methods should be considered to provide appropriate information tailored to the health needs of women with hypercholesterolemia rather than a single educational method that applies to all individuals (7).

## 2. Objectives

The group discussion method, in particular, offers a valuable platform for peer learning, shared experience, and social support, which can be instrumental in fostering motivation and adherence to new behaviors. However, these approaches can be limited by logistical constraints, including scheduling difficulties, geographical accessibility, and the requirement for participants to travel to designated locations. Moreover, the group setting may not be ideal for all individuals, with some women feeling hesitant to discuss personal health information in a public forum.

On the other hand, the advent of mobile technology has opened new avenues for delivering health interventions, offering the potential to overcome some of the limitations of traditional methods. Mobile interventions, particularly those utilizing text messaging, provide a convenient, cost-effective, and scalable approach to health education and behavior change support. This study seeks to rigorously compare the effectiveness of two distinct intervention modalities — group discussion and short message service (SMS) — in promoting positive changes in diet, physical activity levels, and total cholesterol among women with borderline high cholesterol.

## 3. Methods

### 3.1. Study Design and Population

This three-group experimental study (two interventions, one control) investigated middle-aged women (30 - 59) with borderline high cholesterol (200 - 239 mg/dL) at two Kermanshah urban health centers during September 2019. Based on prior research and considering the average changes in cholesterol levels in the three study groups — group 1 (SMS intervention): 22.2 ± 10.89, group 2 (group discussion): 19.2 ± 13.65, and group 3 (control): 16.4 ± 15.4 — with a statistical power of 80% and a confidence level of 95%, the minimum required sample size was calculated to be 55 participants per group using PASS 11 software (8).

**Figure 1.**
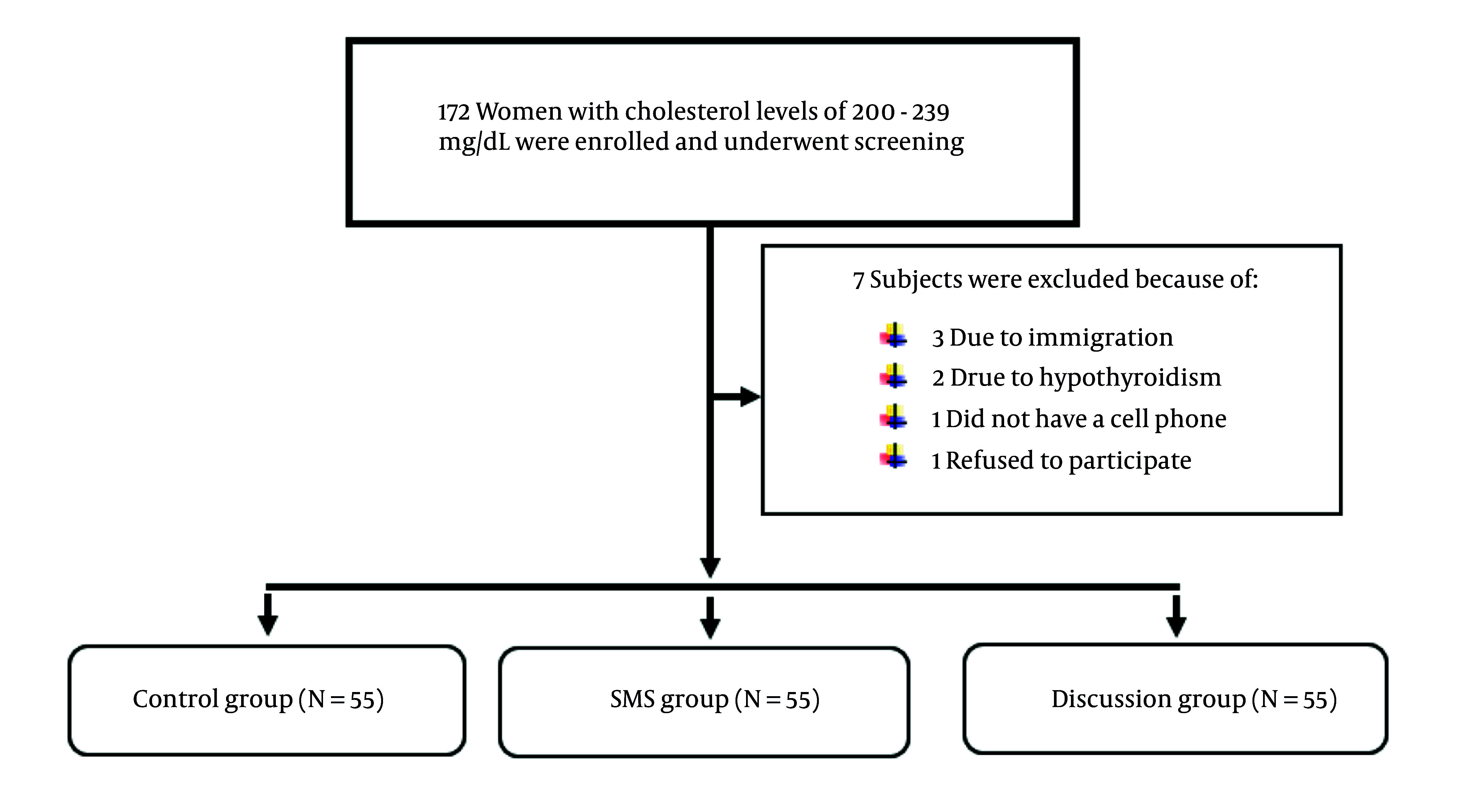
Flowchart of screening, randomization, and intervention

The study attempted to mitigate the influence of confounding variables (age, BMI, and literacy) by creating combined variable categories for adjustment across the various groups. The minimization method was employed for random assignment. The two urban health centers with the greatest population reach were used as the source for sample recruitment. Potential participants were identified from the Integrated Health System (SIB), comprising individuals who had undergone fasting blood sugar (FBS) and cholesterol tests for cardiovascular risk assessment. From the initial pool of 172 individuals contacted, 165 participants were ultimately selected for the study after a thorough review of the inclusion and exclusion criteria. Participants were randomly assigned to one of three groups using permuted block randomization [block sizes of (e.g. 6, 9)], with allocation concealment ensured by sealed, opaque envelopes (Figure 1). 

To participate in this study, individuals had to be between 30 and 59 years old, own a mobile phone, and be able to read text messages. Participants also needed to have borderline high cholesterol levels, specifically between 200 and 239 mg/dL. Individuals were excluded if they had a history of heart disease, diabetes, high blood pressure, hypothyroidism, or were currently taking cholesterol-lowering medication.

### 3.2. Data Collection and Questionnaire

A demographic questionnaire was used to collect data on age, height, weight, BMI, education level, mobile phone ownership, underlying disease status, and medication use. Dietary habits were assessed via the Dietary Habits Assessment Questionnaire. This 11-item questionnaire assessed participants’ consumption of dairy products, type of oil used, and intake of vegetables, fruits, and legumes with dichotomous (e.g. yes/no) response options. The validity and reliability of this questionnaire have been confirmed (9).

The short form of the International Physical Activity Questionnaire (IPAQ) contains 7 questions about physical activity in the past seven days. Physical activities were categorized into three levels of intensity: Vigorous, moderate, and light in this study. These categories were determined based on the sum of each activity over a 7-day period. Activities with a sum exceeding 3000 units were classified as vigorous, those ranging from 600 to 3000 units were classified as moderate, and those below 600 units were classified as light. The units are based on the metabolic equivalent (MET) values assigned to different activities in the IPAQ, multiplied by the duration and frequency of the activities. The IPAQ is also a valid questionnaire and its validity and reliability have been confirmed in Iran (10).

Cholesterol levels were measured using the Sinova D280 autoanalyzer (Sinova Medical Device Co., Ltd., China), which is a reliable and accurate instrument that is widely used in clinical laboratories. To ensure the accuracy and precision of the results, blood samples (2 to 3 cc) were taken from the participants after 12 hours of fasting and analyzed under the same laboratory conditions.

After the educational objectives were defined, the educational content for the three sessions was developed. To ensure content validity, the materials were reviewed by three university professors and two nutrition specialists. Based on their feedback, necessary revisions were made to the content. In the SMS intervention group, participants received three text messages per week, delivered between 6:00 - 6:30 pm every week, with the goal of increasing awareness, providing reminders, and offering encouragement. The content of these messages was limited to 160 characters and focused on topics related to hyperlipidemia, diet, and physical activity. To ensure engagement, participants were instructed to send a confirmation reply (e.g. a specific code) upon receiving each message. Messages were derived from American Heart Association (AHA) guidelines and Iranian Ministry of Health protocols, covering hyperlipidemia awareness (e.g. "Limit saturated fats to reduce cholesterol"), nutrition tips (e.g. "Use olive oil instead of animal fats"), and physical activity prompts (e.g. "Aim for 30 min of daily walking").

Participants in the group discussion were assigned to three groups, each containing 15 - 20 individuals. These groups met bi-weekly at 10 am for three educational sessions lasting 45 - 60 minutes. Pamphlets covering the material of each educational session were provided to participants. Following the distribution of the pamphlets, the researcher gave a short presentation summarizing the key information and then facilitated a discussion among the participants. Attendance was recorded for each session, with an average participation rate of around 95%. Sessions followed an interactive, participant-centered approach, including group discussions on personal challenges in managing cholesterol and motivational techniques (e.g. weekly goal-setting).

These three sessions provided a comprehensive overview of blood lipids, covering key aspects from understanding the different types of lipids and their roles in the body to practical strategies for managing them through diet and exercise. The first session introduced dyslipidemia, the various types of fats, their impact on cardiovascular health, and the importance of a balanced diet as represented by the food pyramid. The second session delved into the specifics of nutrition for individuals with high blood lipids, outlining foods to include and avoid, and explaining how elevated lipid levels affect the heart and blood vessels, increasing the risk of CVDs. Finally, the third session emphasized the crucial role of physical activity in managing blood lipids, detailing how regular exercise can lower levels and improve cardiovascular health, while also providing guidance on choosing appropriate types, intensity, and duration of physical activity. In this study, following data collection, the control group was given an educational booklet comprising the instructed content.

### 3.3. Ethical Considerations

The present study was approved by the Ethics Committee of Kermanshah University of Medical Sciences (IR.KUMS.REC.1397.576). All relevant ethical considerations were observed. The trial was registered at the Iranian Registry of Clinical Trials (IRCT20130812014333N114).

### 3.4. Data Analysis

The data were entered into SPSS version 16, and descriptive and analytical analyses were performed. After checking the normality of the data, the Kruskal-Wallis, chi-square, and Wilcoxon tests were used, and the significance level was considered to be less than 5%.

## 4. Results

In the present study, 165 women aged 30 - 59 with cholesterol levels of 200 - 239 mg/dL participated. Descriptive information, including BMI, age, and education level in all three groups, is given in detail in Table 1. 

**Table 1.** Frequency Distribution of Descriptive Information in the Research Groups ^a^

Variables	Control Group	SMS Group	Group Discussion	Total	P-Value
**BMI**					0.969 ^b^
20 - 24.9	8 (14.5)	8 (14.5)	9 (16.4)	25 (15.2)	
25 - 29.9	28 (50.9)	26 (47.3)	24 (43.6)	78 (47.3)	
≥ 30	19 (34.5)	21 (38.2)	22 (40)	62 (37.6)	
**Age**					1 ^c^
30 - 39	14 (25.5)	15 (27.3)	15 (27.3)	44 (26.7)	
40 - 49	16 (29.1)	16 (29.1)	16 (29.1)	48 (29.1)	
50 - 59	25 (45.5)	24 (43.6)	24 (43.6)	73 (44.2)	
**Education**					0.813 ^c^
Elementary	24 (43.6)	25 (45.5)	22 (40)	71 (43)	
Junior high school	15 (27.3)	12 (21.8)	18 (32.7)	45 (27.3)	
Secondary school	1 (1.8)	3 (5.5)	3 (5.5)	7 (4.2)	
Diploma	15 (27.3)	15 (27.3)	12 (21.8)	42 (25.5)	

Abbreviation: SMS, short message service.

^a^ Values are expressed as No. (%).

^b^ P-value was calculated using Pearson’s chi-square test.

^c^ P-value was calculated using Fisher’s exact test.

The results of the chi-square test showed that there was no significant difference between the three study groups in terms of BMI (P = 0.969), age (P > 0.05), and education level (P = 0.813). Given the non-normal distribution of pre-intervention nutritional status and cholesterol levels across the three groups, the Kruskal-Wallis test was employed for comparative analysis. The three groups exhibited no statistically significant differences in either nutritional status (P = 0.101) or cholesterol levels (P = 0.861) before the commencement of the intervention. Since baseline physical activity levels differed significantly among the three groups (Kruskal-Wallis test, P = 0.02) and were non-normally distributed, absolute changes in physical activity were calculated to assess the intervention’s effect. The results of the Kruskal-Wallis test showed that this difference between the three groups was significant (P < 0.05). Pairwise comparisons revealed that changes in physical activity scores, assessed by energy expenditure, were significantly greater in both the SMS (P = 0.002) and group discussion (P < 0.001) interventions compared to the control group. However, no significant difference in physical activity was observed between the SMS and group discussion interventions (P = 0.24) (Table 2). 

**Table 2.** Mean ± Standard Deviation and Median of the Variables in the Study Groups ^a^

Variables	Groups			
	Control (N = 55)	SMS (N = 55)	Group Discussion (N = 55)	Total (N = 165)
**Nutrition**				
Before	6.27 ± 1.11 (6)	5.94 ± 1.26 (6)	5.72 ± 1.28 (6)	5.98 ± 1.23 (6)
After	6.12 ± 1.1 (6)	7.52 ± 1.19 (7)	8.49 ± 1.05 (9)	7.38 ± 1.48 (7)
**Physical activity (MET-day)**				
Before	299 ± 191 (279)	278 ± 204 (279)	198 ± 222 (9)	258 ± 210 (297)
After	275 ± 153 (279)	339 ± 138 (279)	306 ± 153 (279)	307 ± 150 (279)
**Cholesterol level (mg/dL)**				
Before	216 ± 12 (215)	215 ± 14 (213)	217 ± 15 (216)	216 ± 13 (215)
After	218 ± 12 (218)	205 ± 14 (201)	203 ± 14 (200)	209 ± 15 (206)

Abbreviation: SMS, short message service.

^a^ Values are expressed as mean ± standard deviation (median).

The data presented in Table 3 indicate that both group discussions and SMS interventions yielded statistically significant improvements in physical activity levels relative to the control group (P < 0.001). While nutritional status showed greater enhancement through group discussions (P < 0.001), cholesterol reduction was equally effective across both intervention modalities, with no significant difference between them.

**Table 3.** Findings Related to Physical Activity Index, Nutritional Status Index, and Cholesterol Level Index in the Study Groups ^a^

Variables	Groups			P-Value
	Control	SMS	Group Discussion	
**Physical activity (MET-day)**				
Before	299 ± 191	278 ± 204	198 ± 222	0.02 ^b^
After	275 ± 153	339 ± 138	306 ± 153	0.097 ^b^
P-value	0.041 ^c^	< 0.001 ^c^	< 0.001 ^c^	< 0.05 ^b^
Difference	-24 ± 90	61 ± 121	108 ± 141	
**Nutrition status**				
Before	6.27 ± 1.13	5.94 ± 1.26	5.72 ± 1.23	0.101 ^b^
After	6.12 ± 1.10	7.52 ± 1.19	8.49 ± 1.05	< 0.001 ^b^
P-value	0.262 ^c^	< 0.001 ^c^	< 0.001 ^c^	< 0.001 ^b^
Difference	-0.14 ± 1.06	1.58 ± 0.87	2.76 ± 1.07	
**Cholesterol level (mg/dL)**				
Before	216 ± 12	215 ± 14	217 ± 15	0. 861 ^b^
After	218 ± 12	205 ± 14	203 ± 14	< 0.001 ^b^
P-value	0.307 ^c^	< 0.001 ^c^	< 0.001 ^c^	< 0.001 ^b^
Difference	2 ± 9	-10 ± 4	-14 ± 6	

Abbreviation: SMS, short message service.

^a^ Values are expressed as mean ± standard deviation.

^b^ P-value was calculated using the Kruskal-Wallis test.

^c^ P-value was calculated using the Wilcoxon test.

## 5. Discussion

This study compared the effects of group discussion and SMS interventions on nutritional status, physical activity, and blood cholesterol levels in women with high cholesterol. The results showed that both educational approaches led to significant improvements, suggesting their value in post-screening health programs. These findings are particularly noteworthy because group discussions demonstrated more effectiveness than SMS in improving dietary habits, likely due to their interactive nature, which allows for experience-sharing and immediate feedback (11). Further research is needed to explore the underlying mechanisms through which group discussions lead to greater improvements in nutritional status.

The findings of the present study align with prior research demonstrating the effectiveness of different educational approaches in improving health outcomes. For instance, a comparative study on diabetes self-care revealed that both group-based education and mobile-based education were equally effective, with no significant difference between the two methods (12). While group discussions showed advantages in dietary improvements, the role of SMS-based interventions deserves particular attention, given their scalability and documented efficacy in health promotion. Multiple studies have shown that regular text messaging significantly improves both nutritional practices and physical activity levels among various populations, from hypertensive patients to women visiting health centers, with measurable differences between intervention and control groups (13-17).

Emerging evidence suggests that mobile-delivered education can achieve superior outcomes for certain health behaviors. For instance, one study found that mobile phone-delivered education significantly improved both dietary compliance and overall treatment adherence among hemodialysis patients, whereas face-to-face education, while improving general treatment adherence, showed no significant effect on dietary restrictions specifically (17). However, while studies highlight the potential advantages of mobile-based education, several limitations warrant consideration. Although the use of SMS is recognized as an educational method, its limitations underscore the necessity of leveraging additional mobile phone functionalities, such as interactive games or specialized applications, to enhance the effectiveness and engagement of the educational process.

A comprehensive systematic review and meta-analysis investigated the impact of utilizing mobile applications, both with and without game-based elements, to assess their effectiveness and outcomes. Findings from 36 research studies demonstrated that mobile applications incorporating game-like elements were effective in promoting higher levels of physical activity and contributing to weight loss (18). However, it is important to note that some studies have also shown that digital interventions, including these gamified apps, tend to be more beneficial for men than for women, highlighting potential gender-based differences in engagement and outcomes (19). In contrast, the results of the current study revealed that SMS-based interventions were effective in reducing blood cholesterol levels among women, suggesting that simpler, non-gamified digital approaches may be more suitable for certain demographics.

The present study documented a statistically significant reduction in cholesterol levels following the group discussion and mobile-based educational interventions. A potential reason for the observed reduction in cholesterol levels among women in this study may be attributed to the simultaneous increase in physical activity resulting from the educational content provided during the course of the intervention. Numerous studies have demonstrated the positive impact of increased physical activity on reducing blood cholesterol levels, which is consistent with the findings of the current study (20, 21).

Notably, while the observed 10 - 13 mg/dL reduction in total cholesterol among participants appears modest, its clinical significance becomes evident when considering longitudinal epidemiological evidence. For instance, data from the Framingham study indicate that even a 10 mg/dL decrease in cholesterol is associated with a 2% reduction in CVD risk (22). Furthermore, non-pharmacological interventions typically lower cholesterol by 5 - 20 mg/dL (23). These results demonstrate that even moderate improvements in cardiovascular risk factors can generate significant population-level health benefits when implemented widely. This evidence highlights the preventive medicine value of non-pharmacological approaches to cholesterol management.

Despite efforts to control for confounding variables through rigorous inclusion/exclusion criteria (e.g. age range, health status) and randomization across three study groups, several limitations persist. First, unmeasured confounders such as genetic predispositions, dietary habits outside the intervention, and participants’ adherence to SMS instructions may have influenced outcomes. Second, factors beyond the researcher’s control — including environmental stressors, access to healthcare, or concurrent medications — could not be fully mitigated. While randomization helped balance known and unknown confounders across groups, residual confounding may remain. Future studies could address these limitations by collecting detailed baseline covariates or employing stratified randomization.

### 5.1. Conclusions

The findings of this study highlight the critical role of targeted education in empowering women with high cholesterol to adopt healthier lifestyle practices. The results showed that both educational intervention methods improved nutritional status, physical activity, and reduced cholesterol levels, but this effectiveness was more evident in the group discussion training method, which could be due to various reasons. Therefore, both training methods can be used alone or in combination with the two methods in middle-aged women with high cholesterol. The group discussion training method can lead to better learning in these people due to the creation of active learning, and also the use of short messages as a low-cost and immediate method can be used alongside the group discussion method as a reminder method.

## Data Availability

The dataset presented in the study is available on request from the corresponding author during submission or after publication. The data are not publicly available due to privacy protection and ethical considerations.

## Clinical Trial Registration Code:

The trial was registered at Iranian Registry of Clinical Trials (IRCT20130812014333N114).
